# 2244. What Happened? Antibiotic Resistance During the First Two Years of the SARS-CoV-2 (COVID-19) Pandemic

**DOI:** 10.1093/ofid/ofad500.1866

**Published:** 2023-11-27

**Authors:** Matthew P Crotty, Joel Henderson, Jacqueline Hohulin, Jennifer Wright, Mingyang Cui, Julie Alexander, Leigh Hunter, Mark Hupert, Edward A Dominguez

**Affiliations:** Methodist Dallas Medical Center, Dallas, Texas; Methodist Dallas Medical Center, Dallas, Texas; Methodist Dallas Medical Center, Dallas, Texas; Methodist Dallas Medical Center, Dallas, Texas; Methodist Dallas Medical Center, Dallas, Texas; Methodist Dallas Medical Center, Dallas, Texas; Methodist Dallas Medical Center, Dallas, Texas; Methodist Dallas Medical Center, Dallas, Texas; Methodist Transplant Physicians, Dallas, TX

## Abstract

**Background:**

Increased antibiotic utilization and resistance have previously been demonstrated in the initial wave and surges of the COVID-19 pandemic but there are few evaluations of long-term impact. We sought to assess the utilization of antibiotics and incidence of resistant pathogens prior to and during the first two years of the COVID-19 pandemic.

**Methods:**

Patients age ≥ 18 years admitted between March 2019 – February 2022 were eligible for inclusion. The study timeframe was divided into three phases: pre-pandemic (PRE, March 2019-February 2020), pandemic year 1 (PAN1, March 2020-February 2021), and pandemic year 2 (PAN2, March 2021-February 2022). Antimicrobial utilization (administration) data was summarized as days of therapy per 1,000 patient days (DOT/1000PD). Antibiotic resistance data was summarized as incidence per 100 hospital admissions. Interrupted time-series analysis with linear regression was performed to assess trends in antibiotic utilization and resistance between study time periods.

**Results:**

Significant increases in ceftriaxone (+20.4 DOT/1000PD, P=0.002), azithromycin (+13.3, P=0.002), and anti-pseudomonal β-lactams (APBL; +17.4, P=0.001) utilization were observed between PRE and PAN1 (Figure 1). Subsequently, these increases were followed by significant decreases in utilization from PAN1 to PAN2.

Non-significant increases in the incidence of third-generation cephalosporin resistant bacteria (Figure 2A; +0.71, P=0.171) and carbapenem-resistant bacteria (Figure 2C; +0.34, P=0.068) were observed between PRE and PAN1 (Figure 2).

A non-significant decrease in MRSA slope (P=0.148) was observed from PRE to PAN1 resulting in a reduction in the average monthly incidence of MRSA infections going from 1.09 per 100 admissions in PRE and PAN1 to 0.75 in PAN2 (Figure 3A). A non-significant decrease in vancomycin-resistant enterococci (VRE) incidence per 100 hospital admissions was also observed for VRE over the three year study period (Figure 3B).

**Figure:**
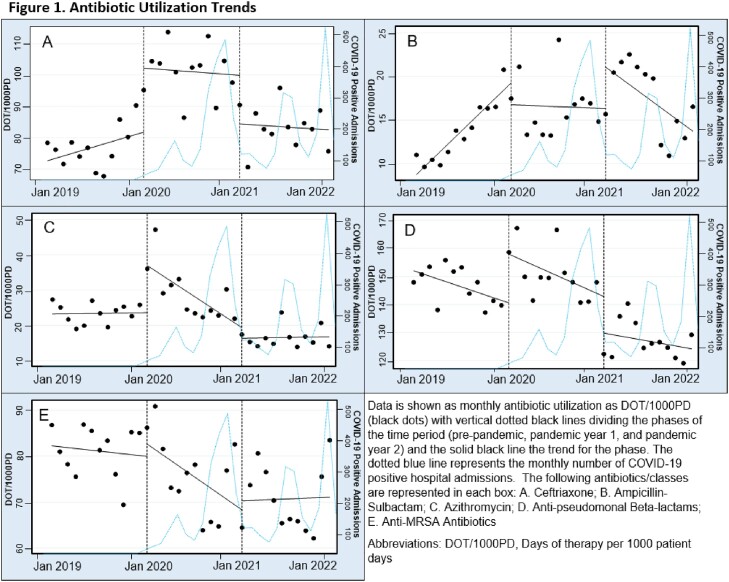

**Figure:**
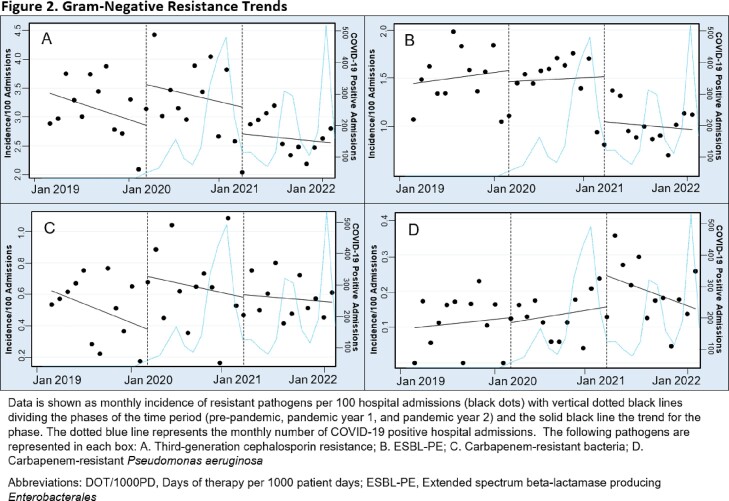

**Figure:**
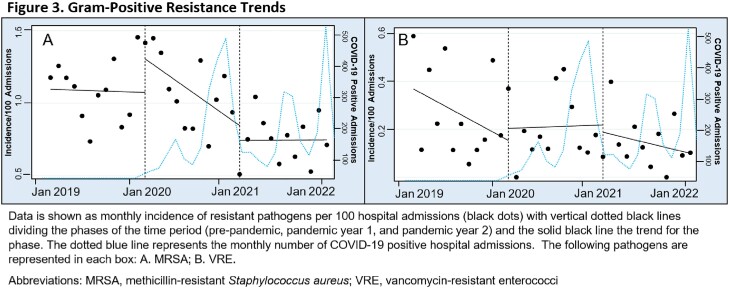

**Conclusion:**

Increased use of key inpatient antibiotics and gram-negative resistance were observed in the first year of the COVID-19 pandemic. Utilization and resistance in PAN2 was similar to that of PRE levels and may represent encouraging signs for the long-term impact on antibiotic resistance.

**Disclosures:**

**Matthew P. Crotty, PharmD, BCIDP**, Paratek Pharmaceuticals Inc.: Grant/Research Support

